# l-Lactate: Food for Thoughts, Memory and Behavior

**DOI:** 10.3390/metabo11080548

**Published:** 2021-08-20

**Authors:** María Fernanda Veloz Castillo, Pierre J. Magistretti, Corrado Calì

**Affiliations:** 1 Biological and Environmental Science and Engineering Division, King Abdullah University of Science and Technology, Thuwal 23955-6900, Saudi Arabia; maria.velozcastillo@kaust.edu.sa; 2 Dipartimento di Neuroscienze “Rita Levi Montalcini”, Università degli Studi di Torino, 10124 Torino, Italy; 3 Neuroscience Institute Cavalieri Ottolenghi, 10043 Orbassano, Italy

**Keywords:** lactate, glycogen, metabolism, behavior, learning

## Abstract

More and more evidence shows how brain energy metabolism is the linkage between physiological and morphological synaptic plasticity and memory consolidation. Different types of memory are associated with differential inputs, each with specific inputs that are upstream diverse molecular cascades depending on the receptor activity. No matter how heterogeneous the response is, energy availability represents the lowest common denominator since all these mechanisms are energy consuming and the brain networks adapt their performance accordingly. Astrocytes exert a primary role in this sense by acting as an energy buffer; glycogen granules, a mechanism to store glucose, are redistributed at glance and conveyed to neurons via the Astrocyte–Neuron Lactate Shuttle (ANLS). Here, we review how different types of memory relate to the mechanisms of energy delivery in the brain.

## 1. Brain Energy Metabolism

The brain represents only 2% of the total body mass, yet to ensure its proper function, it uses between 20 and 25% of the energy produced by the body. This energy consumption is reflected by the use of glucose and oxygen delivered by the blood flow, which represents over 10% of the cardiac output [1]. Glucose is the major energy substrate for mammalian cells; in the brain, it is almost entirely oxidized to CO_2_ and H_2_O through its sequential processing by glycolysis, the tricarboxylic acid (TCA) cycle and the associated oxidative phosphorylation. First, glucose metabolism in astrocytes mainly proceeds through aerobic glycolysis, resulting in lactate production. Lactate taken up by neurons and transformed to pyruvate is then processed through the tricarboxylic acid cycle and the associated respiratory chain [2]. Glucose is also an important constituent of macromolecules and it can be incorporated in glycolipids and glycoproteins present in neural cells. Finally, it may enter the metabolic pathways that result in the synthesis of glutamate, GABA and acetylcholine, key neurotransmitters of the brain [3]. Glucose is also stored in astrocytes in the form of glycogen, a multibranched polymer consisting of thousands of glucose units assembled around a core protein called glycogenin, resulting in various sized granules.

Glycogen is commonly found in the liver, accounting for 6–8%, and skeletal muscle, representing 1–2% of its respective weight. It is also found in the brain, although it only represents about 0.1% of the total brain weight. So the commonly accepted ratio of glycogen in liver, skeletal muscle and brain is 100:10:1 and a variable size of 10 to 80 nanometers in diameter [4,5,6]. Despite its low abundance in the brain, glycogen is the largest cerebral energy reserve and is specifically localized in astrocytes under physiological conditions [7,8]. The glycogen granules act as an energy source under hypoglycemia or ischemia. In the first case, they are able to support energy metabolism by providing a glucose supply for up to 100 min. During ischemia, since no oxidative metabolism occurs, the glycogen stores deplete within two minutes [9,10]. Moreover, it plays a critical role in physiological brain functions such as synaptic activity and memory formation, two conditions requiring a high energy demand [3,11].

**Figure 1:**
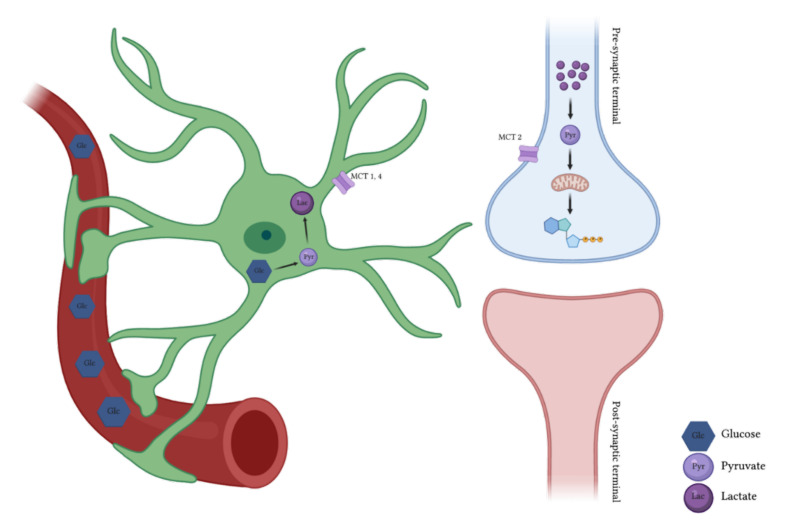
Representation of the astrocyte–neuron lactate shuttle which establishes that in response to glutamate-mediated neuronal activity, astrocytes take up glucose and process it through aerobic glycolysis resulting in lactate formation. Lactate can also be formed through the breakdown of glycogen by increased extracellular K^+^ levels associated with increased neuronal activity or by the activation of noradrenaline and b2 adrenergic receptors [1,17]. Lactate is consequently shuttled from astrocytes via MCT1,4 and taken up by neurons via MCT2 to fuel their tricarboxylic acid cycle.

Glucose metabolism in the brain was first linked to glutamate-mediated neuronal activity through molecular mechanisms based on the role of astrocytes in coupling synaptic activity with vascular glucose intake. This is known as the Neuron-Glia Vasculature (NGV) unit [2,12,13,14,15]. These observations led to the first formalization of the Astrocyte–Neuron Lactate Shuttle (ANLS) model (Figure 1), which states that astrocytes respond to glutamate-mediated neuronal activity by enhancing their level of aerobic glycolysis [16]. Because glycogen is the largest energy reserve in the brain, one of its primary functions is to provide a metabolic buffer during neurotransmission. Under ketogenic conditions, such as breastfeeding, diabetes or starvation, ketone bodies may provide an energy source for the brain [3].

Astrocytes possess the necessary enzymatic machinery for glycogen breakdown and conversion to pyruvate/lactate, which is consequently shuttled to neurons to fuel their tricarboxylic acid (TCA) cycle [3,11]. The metabolism of glucose via glycogen, also known as glycogen shunt activity, has been demonstrated to operate in exercising muscle, as well as in the brain [18,19]. This model establishes that glial glucose flux is divided between glycolysis and glycogenolysis and that the fraction following the glycogenolytic pathway will increase with neuronal activity. This increase in the glycogenolytic pathway allows a rapid neurotransmitter clearance, which will result in a lower oxygen to glucose index, and higher lactate concentrations [19,20]. Lactate is released via monocarboxylate transporters (MCTs) 1 and 4 and taken up by active neurons through MCT 2 to satisfy their energy demands [17]. Recent studies report the crucial role of glycogen metabolism in long-term memory formation, maintenance of long-term potentiation and learning-dependent synaptic stabilization [8,21].

Initially, astrocytes were only considered as non-excitable support cells of the brain, necessary for neuronal distribution and interactions. As the field evolved, it became more evident that astrocytes are necessary to ensure optimal neuronal functioning and communication [22,23]. It is known now, that astrocytes contribute to the morphological remodeling associated with synaptic plasticity, hence acting as spatial and temporal integrators of neuronal activity and plasticity. In fact, synaptic plasticity and memory processes rely on astrocytic regulation of nutrients, i.e., glucose entry to the brain and its metabolism, as well as glycogen accumulation to fulfill high-energy demands [24]. Astrocytes synthetize TCA intermediates needed for the synthesis of glutamate formed by the rapid degradation of glycogen and stimulated by the activation of β_2_-noradrenergic receptors. This, makes the learning process dependent on glycogenolysis and stimulated by noradrenaline [25]. Now, in the context of synaptic plasticity, astrocytes release certain molecules important for this process. Such is the case of TNF-a, which participates in synaptic scaling, a form of homeostatic plasticity that modulates the strength of an entire synaptic network depending on its activity history. Another example is d-serine, which acts as an endogenous NMDA receptor and plays a role in the induction of long-term potentiation in hippocampal synapses [26].

## 2. Memory Systems

Learning and memory are tightly related concepts. In simple terms, learning is the process of acquiring new information; some authors define it as the change in performance as a function of practice. Memory is also the persistence of learning so that it can be recalled later. It is a lasting representation that is reflected in thought, experience or behavior. Both processes demand a wide range of brain areas and involve a series of stages [27,28]. First is encoding, which is a process occurring during the presentation of the learning material. The second stage is storage (also known as retention), which results from encoding and where the information is stored within the memory system. Finally, the third stage is retrieval, which involves recovering or extracting the information previously stored in a particular memory system [29,30].

**Figure 2:**
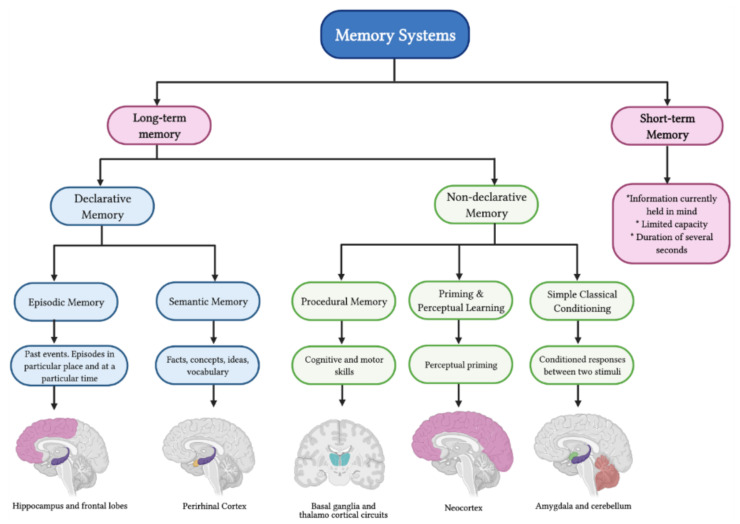
Types of memory and memory systems.

Memory can be classified, depending on the temporal availability of the information, as short-term or long-term (Figure 2). Short-term memory is the memory for information currently held in mind; it has a limited capacity and a duration of several seconds. Long-term memory refers to stored information that does not need to be presently accessed or even consciously accessible. It is considered to have an unlimited capacity essentially, and it can last throughout the lifespan [29,31,32,33]. Nevertheless, this classification gives little reference to the underlying molecular mechanisms or the brain areas involved in this process. For instance, memories can be divided into different categories according to how information is learned, encoded and stored. First, memories are classified either as explicit/declarative or implicit/non-declarative. Declarative memory involves episodic and semantic memory, which refers to the capacity to recollect facts, concepts or ideas, as well as recall particular life experiences or events. This type of memory is representational, providing a way to model the external world. Non-declarative memory is the capacity to recall unconsciously or retain information about automatic learned responses. This type of memory is expressed through performance rather than recollection and includes priming and perceptual learning, procedural memories, simple classical conditioning and non-associative learning (simple reflexes) [34,35,36]. Each of these memories is associated with a particular brain structure, as described below.

Episodic memory refers to the memory of past events in the life of an individual; this involves episodes in particular places at a specific time. These are also known as what, where, when and who or “wwww memories” [34,37,38]. This type of memory allows an individual to re-experience a past event in the context in which it initially occurred, which involves an association of different spatial or non-spatial clues to describe such an event. Therefore, episodic memory requires different brain regions such as the hippocampus and the frontal lobes [35,39]. Several studies show impairments in episodic memory followed by damage to the perirhinal cortex, which has connections to the hippocampus [40].

On the other hand, semantic memory represents information such as facts, concepts, ideas and vocabulary, which is explicitly known and available for recall. This type of memory is usually viewed as an associative network of concepts in which concepts similar to one another are functionally stored together. Some authors even describe it as a concrete and literal “picture memory.” According to classical literature, for semantic memory, the main brain area involved is the perirhinal cortex [34,35,37,41]. More recent studies have shown, particularly analyzing PET scans and fMRI images, reveal that semantic memory is represented by spatially overlapping cortical patterns rather than anatomically segregated regions [42]. Binder and co-workers performed a meta-analysis of 120 studies and concluded that semantic processing occupies a large portion of the cortex and that it could be divided in three broad categories: Posterior heteromodal association cortex (posterior inferior parietal lobe, middle temporal gyrus and fusiform gyrus), subregions of the heteromodal prefrontal cortex (dorsal, inferior, ventromedial prefrontal cortex) and medial paralimbic regions (parahippocampus and posterior cingulate gyrus) [43].

Procedural memory is the kind of memory that stores processes, allowing the ease of performing specific activities or cognitive operations; this may include stimulus-response associations. In this case, the information is learned unconsciously as a skill, it can also be difficult to explain verbally and the memory persists for a long time [41,44]. Procedural memory can be further subdivided into motor, perceptual or cognitive. Examples of these are speech production, riding a bike, typing on a keyboard, swimming, walking, playing golf and driving a car. Procedural memory mainly depends on the basal ganglia to encode and consolidate an event; it involves complex and collective synaptic firings in the frontal–basal ganglia–thalamocortical circuits [29,33]. A study carried out in 2008 showed irregular striatum activity in an obsessive-compulsive disorder model, where procedural memory tasks are often confused, emphasizing the importance of the basal ganglia in this type of memory [45].

Priming and perceptual learning can be described as a technique by which a priming stimulus is used to sensitize the neuronal representation of the stimulus to train for a later presentation of that or a similar stimulus [29,33]. Several studies show that this type of memory is preserved in patients with amnesia [34,35] and that priming facilitates perceptual processing [46]. Priming effects can be perceptually, conceptually or semantically driven [29,47]. While this type of memory is only expressed in performance and cannot be reflected in a verbal report, its effects can be associated with declarative and procedural memory and it may depend on the neocortex. Notably, pavlovian conditioning relies on this type of memory [34,35].

The concept of classical conditioning was first mentioned in the early 1900s as a means of studying associative learning. Classical conditioning is an associative memory between a conditional stimulus and unconditioned responses, such as rewards and punishments. In this way, two stimuli (one that naturally produces a response and a neutral one) are presented together to produce a new learned response; after repeated pairings, the neutral stimulus alone will elicit the response. Therefore, classical conditioning studies the relationship between the stimuli and the environment [29,33,48]. When the conditioned stimulus triggers an emotional response, the amygdala is the brain area involved in this process [35]. Studies carried out in monkeys and rats show that amygdala lesions produce a lack of emotional response, excessive examination of objects and an incorrect pairing of food rewards, demonstrating an impairment in the processing of reward-related stimuli [40]. If the conditioned stimulus results in a skeletal response, the cerebellum is the brain area involved [35]. These findings were supported by multiple classical eyeblink conditioning studies, a valuable experiment for analyzing the behavioral and neuronal aspects of acquisition and retention of learned responses [49,50].

In recent years transgenic knockout mice have been used to carry out classical conditioning studies; this has made it possible to study changes in hippocampal synapses [51]. Additionally, neural recordings of the cerebellum during eyeblink conditioning in a rabbit show increased and decreased extracellular activity in the dentate/interpositus deep nuclei and cortex. These results are correlated with the conditioned stimulus and response, as well as unconditioned stimulus and response. It was also found that the excitability of Purkinje cells is highly correlated with the acquisition of a conditioned response [48]. Several models propose cerebellar plasticity at the synapses between the parallel fibers and the Purkinje cells, resulting from the activation of mossy and climbing fibers [50].

### Molecular Mechanisms behind Memory

Memory retention is the process in which acquired information is transformed into a stored mental representation that is maintained over time without needing an active rehearsal [29]. In addition, memory consolidation is a progressive stabilization of long-term memory traces so that they become relatively resistant to decay or disruption. Memory consolidation is divided between rapid (synaptic) consolidation and system consolidation. The first one is accomplished within the first minutes to hours after learning and involves gene transcription and protein formation, leading to lasting cellular channels to support long-term memory. System consolidation can take from days to years to complete and involves the interaction between the medial temporal lobe and the neocortex [28]. These processes require neurotransmitter receptors for the acquisition and storage of new memories [36]. Examples of these are N-methyl-D-aspartate (NMDA) [52], Adenosine A2A [53], dopamine Drd1a [54], AMPA, GABA and metabotropic glutamate receptors [36], as well as acetylcholine, serotonin and norepinephrine [55,56]. It has been established that during the consolidation process, the medial temporal areas play a critical role; this system undergoes several functions related to memory, such as encoding, consolidation and retrieval. Eventually, memories will become independent of the medial temporal area and will depend on specific neocortical regions [33,57].

Changes in synaptic strength underlie memory storage and other adaptive responses, including pain control, mood stability and reward behavior. Synaptic consolidation indicates the development and stabilization of protein synthesis-dependent modifications in synaptic strength to support long-term memory formation and maintenance. This process is observed during long-term potentiation (LTP) and long-term depression (LTD). Additionally, synaptic consolidation requires brain-derived neurotrophic factor (BDNF) signaling and the immediate early gene activity-regulated cytoskeleton-associated protein Arc [28,58].

LTP is considered a neural mechanism essential for synaptic plasticity and it is the most common mechanism underlying associative learning. LTP can be evoked by high-frequency stimulation (HFS), which results in the long-lasting enhancement of synaptic efficacy. Moreover, activation of NMDA receptors is sufficient for inducing LTP, since it has been demonstrated to be the molecular substrate of the process [51,52].

BDNF triggers synaptic consolidation in mature excitatory synapses through its tyrosine kinase receptor (TrkB); this process is carried out in two stages. First, the translation stage is where high-frequency stimulation leads to the post-synaptic release of BDNF and the activation of TrkB receptors, present in pre- and post-synaptical elements of glutamatergic synapses. Particularly, post-synaptic TrkB receptors rest in the post-synaptic density (PSD) while TrkB co-immunoprecipitates are found in the NMDA receptor protein complex. Second is the Arc dependent consolidation stage; Arc encodes the only mRNA known to undergo transport to distal dendritic processes of granule cells [58].

Hippocampal studies show that Arc mRNA is enriched at stimulated synapses and Arc protein is elevated in dendrites following LTP induction. This sustained translation of Arc is crucial for cofilin phosphorylation, local F-actin expansion and the formation of stable LTP [58]. During behavioral training, Arc is expressed in principal neurons, which is necessary for long-term spatial memory [59,60]. Interestingly, a recent study in the primary visual cortex demonstrates that Arc protein in spines increases in LTD and decreases in LTP. The authors of this study conclude that Arc helps organize the distribution of potentiated and depressed spines, which underlies the plasticity of neuronal responses [61].

The synthesis of new proteins, i.e., mRNA translation, is critical for memory formation and long-lasting synaptic plasticity and for reconsolidation. This process can be triggered by gene expression changes, learning-induced activation of neuronal receptors, intracellular signaling pathways or epigenetic mechanisms [62]. Some transcription factors that participate in these tasks are cAMP Response Element–Binding Protein (CREB), C/EBP, AP1, c-Fos, Zif268, NFkB, activating transcription factor [ATF-4] [63,64]. They can bind to DNA response elements such as CRE to regulate RNA polymerase activity and determine the time and level of gene expression. Moreover, the CREB1 acts as a transcriptional activator after its phosphorylation by kinases such as PKA, MAPK, CamKIIa [65,66]. In addition to the previously mentioned transcription factors, nuclear factor kappaB (NF-kB), serum response factor, junB and neuronal Per-Arnt-Sim homology factor 4 (NPAS4) also play important roles in the memory consolidation process [64,67,68]. Protein synthesis in neurons occurs in the dendrites; this allows a rapid and precise localization of protein expression in response to synaptic activity and also provides a critical mechanism for synaptic formation, maintenance and plasticity [62].

A clear example of this is mTOR, a protein that integrates inputs from several signals such as activation of neurotransmitters, growth factor receptors and cellular metabolism changes. This protein acts together with TORC complex 1 or 2 (mTORC1, mTORC2) to regulate protein synthesis and cell growth or to control cell cycle progression and energy metabolism [62]. Several studies have shown the important role of mTORC1 in synaptic plasticity and memory reconsolidation. Findings in rat hippocampal slices, for example, show that the disruption of mTOR signaling reduces late-phase LTP expression induced by HFS without affecting early phase LTP and also blocks the synaptic potentiation induced by BDNF [69]. The idea that mTOR signaling is required to form and reconsolidate long-term memory is further supported by behavioral studies that tested spatial memory formation [70], object recognition memory [71] or involving fear-motivated tasks [72]. These studies will be discussed in detail in the following sections of this review.

Leptin receptors (LepR) in hippocampal astrocytes have also been demonstrated to play an essential role in synaptic transmission, plasticity and brain metabolism [73,74,75]. In one study, Naranjo et al. used a genetic mouse model that lacked the expression of LepR in GFAP-positive cells. They evaluated synaptic transmission and hippocampal plasticity using electrophysiological recordings and assessed the expression of enzymes and transporters involved in glutamate metabolism. Their findings confirmed that LepR in astrocytes are involved in maintaining glutamate homeostasis and neurotransmission since LepR depletion reduced basal synaptic transmission in CA1 cells and impaired NMDA-LTD. In addition, genetically modified mice exhibited lower glutamate uptake efficacy and upregulation of GLUT-1, GLT-1, GFAP and glutamine synthase, which could impact learning and memory processes [76].

## 3. Lactate: A Key Molecule for Memory

Brain energy is crucial to support the action potentials required for neuronal communication, maintenance of ionic gradients across the plasma membrane, protein synthesis, phospholipid metabolism or neurotransmitter recycling [3]. The astrocyte–neuron lactate shuttle establishes that presynaptic glutamate released from excitatory boutons is taken up by astrocytes; this glutamate is then recycled as glutamine (glutamate-glutamine cycle) and further released from astrocytes to neurons to form new glutamate for vesicle storage. Glutamate is taken up with Na^+^ through specific astrocyte transporters, resulting in a dissipation of the Na^+^ gradient which are reestablished through the activity of the Na/K-ATPase [77]. Both Na/K-ATPase activity and glutamine formation from glutamate are highly energy consumptive processes; astrocytes increase the glucose uptake from the bloodstream. Surprisingly, instead of using glucose through oxidative phosphorylation in mitochondria to produce ATP, they use the glycolysis pathway to produce a few ATP molecules; this process (also known as “aerobic glycolysis” or “Warburg effect”) is accompanied by the synthesis of lactate, which is released via MCT1 and MCT4 and taken up by active neurons through MCT2. In neurons, lactate is transformed into pyruvate and is subsequently metabolized through oxidative phosphorylation, yielding between 14 and 17 ATPs per lactate molecule [12,78]. Lactate shuttle from astrocytes and further uptake by neurons play an essential role in learning, memory consolidation and LTP [8,21,79,80].

One such study to confirm the importance of ANLS in LTP and hippocampal memory formation was performed by Suzuki and collaborators using electrophysiological and behavioral experiments. The electrophysiological studies showed that LTP could be triggered in CA1 neurons following Schaffer collateral stimulation with the increased fEPSP slope, a classic indication and monitor of increased synaptic efficacy. Behavioral trials were conducted on rats performing the inhibitory avoidance test. Rodents received a bilateral hippocampal injection of 1,4-dideoxy-1,4-imino-d-arabinitol (DAB), which is a glycogen phosphorylase inhibitor. Researchers found that DAB prevented LTP maintenance and hypothesized that the intrahippocampal application of additional lactate could bypass it. These findings indicate that neurons require lactate uptake to meet the energy demands of LTP induction, even when displaying average concentrations of glucose. Therefore, lactate should be available for neurons during the conditioning phase of the behavioral test. Results show that the application of lactate after conditioning does not restore LTP. This indicates that ANLS plays a critical role in long-term synaptic plasticity, long-term memory, as well as molecular and synaptic changes [21].

A similar study by Duran et al. examined the learning capacities and electrophysiological properties of the hippocampal CA3-CA1 synapse using glycogen synthase knockout mice. The electrophysiological results show that paired-pulse facilitation (a form of short-term plasticity related with short-term memory) is enhanced in the mice lacking glycogen synthase. Moreover, paired-pulse stimulation (an indirect measurement of the probability of neurotransmitter release) reflects a disturbance in the release of neurotransmitters at the presynaptic terminal in the knockout mice. This confirms the role of glycogen as a precursor of glutamate and its importance in short-term memory processes. Finally, the knockout mice did not show significant LTP after the stimulation session, suggesting that glycogen is a crucial energy source to evoke this change in synaptic strength. The authors also conducted a behavioral test using the Skinner box. The results from his test reveal a significant impairment in the learning process of mice lacking glycogen synthase, which supports the previous results [81].

To support the idea that lactate regulates synaptic potentiation at central synapses and contributes to the process of memory formation, Herrera-López and co-workers carried out a series of electrophysiological experiments on hippocampal slices. They demonstrated that extracellular lactate induces glutamatergic potentiation on the recurrent collateral synapses of hippocampal CA3 pyramidal cells (CA3 PC). This potentiation occurs through a post-synaptic lactate receptor mechanism, calcium accumulation and NMDA receptor activation. The researchers found that lactate does not induce potentiation at the mossy fiber synapses of CA3 PC, concluding that lactate triggers an input-specific form of synaptic plasticity on the hippocampus and that it increases the output discharge of CA3 neurons when recurrent collaterals are repeatedly activated during lactate perfusion [82].

The degree to which long-term modifications in synaptic strength are complemented by modifications in lactate dynamics is still a matter of research. To understand it, Bingul et al. induced LTP of synapses in the dentate gyrus in freely behaving rats; this process was done through HFS of the medial perforant pathway. Before, during and up to 72 h after LTP induction, the extracellular lactate concentrations were measured using fixed potential amperometry, allowing the evaluation of how changes in synaptic strength modify local glycolytic activity. They found that synaptic potentiation was associated with persistent alterations in acute lactate dynamics following neuronal activation and observed chronic lactate availability within the dentate gyrus. These changes in lactate dynamics were only visible 24 h after HFS, whereas synaptic potentiation and altered lactate dynamics lasted up to 72 h. The authors conclude that these observations reflect a metaplastic effect that could regulate the memory consolidation process. Furthermore, these changes in extracellular lactate concentrations could support the increased energetic demands or play a neuroprotective role [83]. In order to monitor lactate dynamics Mächler and co-workers used a genetically encoded FRET sensor in combination with in vivo two-photon laser scanning microscopy. Following opening of MCTs in astrocytes and neurons using a transactivation process, they observed at first a decrease in lactate signal in astrocytes followed by an increase of it in neurons, demonstrating a lactate gradient between these two cell types that favor the flow of lactate from astrocytes to neurons, consistent with the ANLS [84].

The ANLS model establishes that lactate is released from astrocytes through MCT1 and MCT4 and taken up by neurons through MCT2, which makes these transporters critical in learning and memory formation [17]. To better understand their role, Netzahualcoyotzi and Pellerin used transgenic mice and a viral vector to decrease the expression of each transporter within the dorsal hippocampus. They demonstrate that both neuronal MCT2 and astroglial MCT4 are essential in spatial information acquisition and retention in different hippocampal-dependent tasks. After an intracerebral injection of lactate, mice with reduced levels of MCT4 exhibited improved spatial memory, but this manipulation did not affect mice with an MCT2 knockdown, supporting the idea that ANLS contributes to hippocampal-dependent learning. In contrast, MCT2 is shown to be required for long-term memory formation seven days after training, and plays an important role in mature neurons in the process of adult neurogenesis in the dentate gyrus [85].

Long-term memory formation is also affected by the release of noradrenaline and β-adrenergic signaling, which occurs in states of arousal because the coeruleo-cortical noradrenergic projection, results in noradrenaline release in the cortex. Noradrenaline has been shown to trigger glycogenolysis in astrocytes [86] resulting in aerobic glycolysis, consequently stimulating lactate production from glycogen [87]. Fink and collaborators studied single noradrenaline-stimulated astrocytes by measuring cytosolic lactate concentration using a FRET nanosensor; this process was done under different pharmacological conditions. First, they used 2-deoxy-d-glucose, a non-metabolizable form of d-glucose, to interfere with lactate metabolism; second, DAB, a potent inhibitor of glycogen phosphorylase and glycogen degradation; and finally, 3-nitropropionic acid (3-NPA), an irreversible inhibitor of succinate dehydrogenase, a Krebs cycle enzyme. Their findings reveal that d-glucose uptake is critical for the noradrenaline-induced increase in lactate concentration resulting from glycogen degradation, suggesting that most glucose molecules in the noradrenaline-stimulated cells transit through a glycogen shunt. In addition, it was observed that under these pharmacological conditions and a defined transmembrane glucose gradient, the glycolytic flux intermediates are used to produce lactate and support oxidative phosphorylation via pyruvate. This was demonstrated by an increase in lactate concentration during inhibition of the Krebs cycle [88].

To confirm the role of noradrenaline in lactate production, Zuend et al. investigated lactate dynamics in neurons and astrocytes in awake mice. They exposed the mice to isoflurane, which caused a strong arousal response, pupil dilatation and Ca^2+^ elevations in both neurons and astrocytes. These alterations in cortical activity triggered an extracellular lactate release which correlates with a fast and prominent lactate dip in astrocytes, followed by a delayed rise in neuronal and astrocytic lactate [87]. The work by Gao and collaborators also illustrates the role of adrenergic signaling in modulating long-term memory consolidation by activating glycogenolysis and subsequent lactate release [89]. These changes altogether suggest activity-dependent glycogen mobilization and further lactate release from astrocytes, which are critical in the long-term memory formation and consolidation processes [84,87,89].

Lactate also plays an important role in supporting the expression of genes such as Arc, c-Fos, Bdnf and Zif268, which involved in plasticity and neuronal activity [90]. Yang and co-workers investigated this matter in vitro in primary cultures of neurons and in vivo in the mouse sensory-motor cortex. They found that lactate stimulates the expression of genes such as Arc, c-Fos and Zif268, which are related to synaptic plasticity, and that these effects were not replicable with glucose nor pyruvate. This upregulation is carried out through a mechanism involving NMDA receptor activity and its downstream signaling cascade Erk1/2. The researchers found that lactate potentiates NMDA receptor-mediated currents, which produces elevated intracellular calcium via an increased calcium influx. Furthermore, lactate increases the intracellular levels of NADH associated with changes in the redox state of neurons. NADH mimics the effects of lactate on NMDA signaling, leading to the idea that an increase in NADH directly affects the effects of lactate [91]. In another study Margineanu and collaborators used RNA-sequencing to identify synaptic plasticity promoting genes. In addition to those found by Yang et al., they identified that Erg2, Erg3, Erg4, Npas4, Nr4a3 and Rgs4 are modulated by L-lactate in cortical neurons. Moreover, they identified ten genes associated with the MAPK signaling pathway; those are: c-Fos Bdnf, Atf4, Nr4a1, Gadd45g, Map3k11, Dusp4, Dusp6 and Dusp10 [92]. These studies lead to the conclusion that lactate can be considered a signaling molecule in neuronal plasticity, in addition to its role in energy metabolism.

### The Role of L-Lactate in Disease

Lactate production in astrocytes and its sequential shuttle to neurons is an essential process in learning, memory consolidation and LTP. Accordingly, anomalies in the brain energy metabolism can result in severe pathologies or aggravate pre-existing conditions. In particular, Alzheimer’s Disease (AD), amyotrophic lateral sclerosis (ALS), depression, stress and schizophrenia show disruptive lactate signaling between astrocytes and neurons [93]. For instance, Positron Emission Tomography (PET) scans have documented reduced glucose utilization in brain regions affected by patients with Alzheimer, Parkinson and Huntington’s disease, as well as with ALS [94].

AD is one of the most common forms of dementia. In its preclinical stage, brain glucose hypometabolism is recognized as a prominent anomaly and some studies suggest that glycogenolysis plays a critical role in the development of the disease [95]. Impairments in glycogen synthesis could reduce glycogen levels, impeding the physiological flux of glucose units through glycogen, consequently affecting learning and memory processes [96]. Research shows reduced levels of GLUT1 and GLUT3, which correlates with less glucose uptake, which translates into a subsequent cognitive decline. Furthermore, the enzymatic activity of phosphofructokinase, phosphoglycerate mutase, aldolase, glucose-6-phosphate isomerase and lactate dehydrogenase display a loss of activity in patients with AD in comparison with age-matched controls [94]. Ryu and collaborators compared neural progenitor cells and astrocytes differentiated from late-onset AD patients. The authors found a significant downregulation of lactate dehydrogenase A in both cell types and that astrocytes from late-onset AD have a reduced metabolism of lactate [97].

In the case of Parkinson’s Disease (PD), glucose hypometabolism has been documented. Key enzymes glucose-6-phosphate dehydrogenase and 6-phosphogluconate dehydrogenase are expressed in lower levels in putamen and cerebellum of PD patients [94]. Other studies show an increase in lactate levels in the striatum of patients and animal models of advanced PD [98,99].

On the other hand, ALS is characterized in patients by loss of motor neurons in the brain and spinal cord, as well as glucose intolerance, insulin resistance and hyperlipidemia. At the cellular level is common to find altered endothelial transporter proteins and astrocyte end feet degradation [94]. Nonetheless, research has shown that lactate could be used directly as cerebral uptake or indirectly as gluconeogenic precursor to improve ALS symptoms [100,101].

Schizophrenia and bipolar disorders are common and severe psychiatric disorders. They characterize by overlapping genetic background, brain abnormalities and clinical presentations. Some research suggests that alterations in brain metabolism and mitochondrial function are evident in these disorders. A set if studies ex-vivo using mouse models of schizophrenia, bipolar disorder and autism spectrum disorders showed lower pH and higher lactate levels in all the models [102]. In vivo studies in animal models and in patients confirm this evidence. Lactate concentrations are elevated and negative correlated with general cognitive function and functional capacity [103,104,105]. In contrast, patients suffering from depression can benefit from lactate as a treatment option. It has been proved that lactate administration produces antidepressant-like effects, promotes resilience to stress and rescues social avoidance and anxiety behaviors [106,107].

## 4. Behavioral Perspective

Most of the studies presented previously provide evidence that supports the importance of brain energy metabolism in learning and memory processes. This section aims to describe how behavioral studies enlighten our knowledge on brain energy metabolism in particular types of memories.

### 4.1. Spatial Memory

Spatial working memory is mediated by astrocytic glycogenolysis and by the expression of synaptic plasticity promoting genes [79,108,109]. To better understand this, Newman, Korol and Gold used a spontaneous alternation task using the plus-shaped maze in behaving rats. For this experiment glucose or lactate-sensitive biosensor was used to measure glucose and lactate levels in extracellular fluid in the rat hippocampus before, during and after memory tests. The recordings from the biosensors revealed a significant increase in lactate concentrations at the beginning of the behavioral test. Then, glucose levels dropped 5 min after initiating the task and 5 to 10 min while performing the test, the glucose levels raised again; these changes could correspond to an increase in blood glucose levels. After completing the task, a significant increase of lactate was again recorded; it is believed that this is a consequence of handling after removing the mice from the maze. Additionally, a pharmacological inhibition of astrocytic glycogenolysis and a pharmacological block of MCT2 by a hippocampal injection of α-cyano-4-hydroxycinnamate, resulted in memory impairment. In the first case, lactate or glucose administration was sufficient to reverse this effect; nevertheless, either glucose or lactate were able to restore the memory impairment caused by the block of MCT2 [79].

To highlight the importance of MCTs in the ANLS, Ding and co-workers established a model of long-term ketamine administration aiming to examine changes in MCTs expression that will lead to learning and memory deficits. In this case, mice were exposed to intraperitoneal administration of ketamine for six months; long-term ketamine administration is associated with abnormalities in MCTs that cause hippocampal dysfunctions. During the ketamine-administration period, mice were trained and tested for the Morris water maze (MWM) to assess their spatial memory performance and for the Radial arm maze (RAM) to evaluate their spatial working memory performance. The authors report that mice exhibited learning and memory deficits. When quantifying hippocampal proteins, the membrane fraction showed a significant decline of MCT1 and MCT4 proteins, whereas the cytoplasmic fraction showed increased levels of MCT1 and MCT4. Moreover, the global expression of MCT2 was enhanced. Finally, mRNA analysis showed that the expression on MCT2 mRNA was significantly increased, whereas MCT1 and MCT4 transcripts displayed no changes. Supposedly, cognitive deficits observed in the behavioral tests were related to the reduced levels of hippocampal membrane MCT1 and MCT4 [110].

As previously mentioned, mTOR plays an important role in regulating protein-synthesis-dependent synaptic plasticity and memory formation [62]. Dash, Orsi and Moore investigated the role of mTOR in long-term spatial memory formation using the MWM. The researchers administered either rapamycin (mTOR inhibitor), glucose, 5-amonoimidazole-4-carboxamide-1-B-4-ribonucleoside (AICAR; AMP kinase activator) or a mix of glucose and rapamycin into the dorsal hippocampus of Long-Evans rats after training in the MWM. The results suggest that AICAR and rapamycin impair long-term spatial memory, whereas glucose improves it. Moreover, the authors aimed to examine a potential mechanism to restore memory impairment by the co-administration of glucose and rapamycin; however, in this case, memory impairment was not reversed [70].

Learning and memory retrieval are both energetically demanding processes. In order to explore the role of lactate production in these processes Harris and colleagues injected dichloroacetate (DCA) into the frontal cortex and hippocampus of mice. DCA is a chemical inhibitor of lactate production; it inhibits pyruvate dehydrogenase (PDH) kinase by enhancing the activity of PDH, which further attenuates the conversion of pyruvate to lactate. The authors examined the effect of DCA on spatial learning and memory, which requires communication between the frontal cortex and the hippocampus. For this, they used the MWM as a behavioral task. The results were obtained by in vivo 13C-pyruvate magnetic resonance spectroscopy, revealing a decrease in pyruvate conversion to lactate after the DCA administration, which was accompanied by a reduction in the phosphorylation of PDH. The behavioral studies showed impaired learning in those mice injected with DCA 30 min before training, which resulted in memory impairment during the probe trial. In contrast, mice that received the DCA injection before the probe trial and not before training exhibited a standard memory. When testing memory retrieval using the MWM, the researchers found that DCA administration does not significantly affect the recall of established memories, even four days after training. These findings suggest that aerobic glycolysis, and hence lactate production, are required for memory acquisition but not for retrieval [111].

### 4.2. Object Recognition Memory

To understand the role of BDNF in the rat hippocampus Radiske and collaborators used the novel object recognition (NOR) test. For this, rats were trained in NOR with two different stimuli objects; 24 h later, they were undergoing a refresh session for five minutes using a familiar and a novel object. After memory reactivation, rats received a bilateral injection in the CA1 area of the hippocampus; they received either a vehicle or an anti-BDNF antibody. Then, the animals were exposed to a familiar and a novel object for five minutes to evaluate long-term memory retention. The authors found that reactivation in the presence of a novel object destabilizes object memory recognition to initiate reconsolidation in the hippocampus. These results indicate that BDNF is sufficient for controlling the integration of new information into the memory system and that object recognition memory retrieval increases BDNF levels in dorsal CA1. Finally, the amnesia caused by mRNA and protein synthesis inhibitors can be reversed by BDNF signaling reactivation following memory refresh [112].

l-Lactate plays a role as a metabolic and signaling molecule, accordingly, Vaccari-Cardoso and co-workers developed a viral vector to express a modified version of lactate oxidase (LOx) originating from the bacteria Aerococcus viridans. Their results in vitro show that LOx expression in astrocytes reduced their intracellular lactate levels and its release to the extracellular space. The researchers used the hole board test to measure exploratory behavior and they observed that mice expressing LOx in hippocampal astrocytes manifested an increased activity compared to control mice. Mice expressing LOx exhibited improved performance in the Y-maze task, which tests spatial recognition memory, but not in the Y-maze spontaneous alternation task or the NOR test. They concluded that a selective decrease in intracellular lactate pool in hippocampal astrocytes contributes to increased responsiveness to novel stimuli [113].

To elucidate the role of the basolateral complex of the amygdala (BLA) in recognition memory, Jobim et al. used the NOR task in Wistar rats. The researchers compared the effects of mTOR inhibition by rapamycin infusion into the BLA or dorsal hippocampus; this was done before or after training or reactivation. Results show that rapamycin infusion, either before or after training, impairs NOR retention tested 24 h after training. In particular, memory retention is impaired when the infusion is given before reactivation on BLA or dorsal hippocampus and measured 24 h after the reactivation, but this does not occur if measured six hours after reactivation. These findings indicate that mTOR signaling is crucial for the consolidation and stabilization of object recognition memory, either in the hippocampus or BLA. mTOR acts as a regulator of glucose uptake, glycolysis, lipid synthesis and mitochondrial metabolism; consequently, its inhibition might influence neuronal metabolism, which will affect memory-modulatory function [71].

Continuing with the exploration of different brain areas involved in object recognition memory, Korol and colleagues evaluated the involvement of lactate in the hippocampus and striatum. This test was performed with Long-Evans rats and recognition memory was assessed using the double object location (DOL) task and the double object replacement (DOR) task. Rats received an injection of lidocaine (Na^+^ channel blocker) and 4-CIN (MCT2 inhibitor) into either hippocampus or striatum after three training sessions and before the test trial. Findings demonstrate that both lidocaine and 4-CIN impair recognition memory for objects and their relative location; this only occurs when the substance is administered to the particular brain area necessary for that type of recognition. Infusion into the hippocampus impairs the recognition in the DOL task, whereas the ones in the striatum impaired the recognition in the DOR task. In conclusion, neuronal lactate uptake in both hippocampus and striatum is necessary for object recognition memory [80].

### 4.3. Fear Conditioned Memory

Noradrenaline acts through adrenergic receptors, of which β-adrenergic receptors (β -AR) in the amygdala or hippocampus play crucial roles in encoding and consolidating memories, particularly fear related memories. Noradrenaline activates glycogenolysis and consequently lactate release, which is critical in memory processing [88,89,114,115]. With this in mind, Gao et al. used the hippocampus-dependent IA task to determine if astrocytic or neuronal β-ARs in the hippocampus mediate memory consolidation. Their results show that astrocytic ARs (β2-AR) play a critical role in the consolidation of a fear-based contextual memory. Moreover, β2-AR mediates learning-dependent lactate release from astrocytes, which is necessary to support the molecular changes needed for long-term memory formation [89].

Noradrenergic innervation to the cortex originates from neurons in the locus coeruleus and this system plays a key role in the sleep wake cycle, arousal, respiration, learning and memory [115]. To investigate how noradrenergic activity modulates Ca^2+^ and cAMP dynamic during fear conditioning, Oe and co-workers imaged astrocytes in the auditory cortex of behaving mice. First, they tested if a startle response increases Ca^2+^ and cAMP levels; to do so, mice received unpredictable air puffs on the right side of the face. As a result, Ca^2+^ was elevated, but no significant cAMP increase was recorded. Then, the researchers examined astrocytic activity during fear memory acquisition. In this case, the mice had their head fixed to allow imaging of the cortex throughout the experiment and they received a foot shock after a sound cue. During recall on the next day, only the sound cue was presented and the establishment of the fear memory was manifested by increased immobility times. The results of this second experiment showed that foot shock induces and elevates both astrocytic Ca^2+^ and cAMP, although it is attenuated with repeated shocks. Finally, the authors conclude that these changes might be involved in the modulation of synaptic transmission and memory consolidation [116].

To test if the supply of glycolytic metabolites such as pyruvate or β-hydroxybutyrate can functionally replace lactate in a memory impairment model Descalzi and collaborators performed a series of experiments. First, they used the IA task in adult rats and injected DAB into the dorsal hippocampus to generate memory impairment. After the DAB injection, they observed that it reduced the latency of rat entry into the shock compartment postconditioning; nevertheless, this latency was increased by a co-injection of DAB and either pyruvate or β-hydroxybutyrate. Then, they performed an expression knockdown of MCT1, MCT2 and MCT4 that resulted in a reduced latency post-conditioning. The supply of pyruvate and β-hydroxybutyrate counteracted this effect and rescued the memory loss caused by the knockdown of MCT1 and MCT4, but it did not affect the decrease in latency in the MCT2 knockdown. In conclusion lactate is critical in providing energy for neuronal responses required in long-term memory. The authors suggested that learning and training increase mRNA translation expressed in excitatory and inhibitory neurons, which can be blocked by inhibiting glycogenolysis and rescued with a co-injection of DAB and lactate [117].

To assess the role of mTOR signaling in long-term memory, Beckinschtein and co-workers performed a one-trial IA test in rats. First, the authors demonstrate that IA training is associated with rapid increases in the phosphorylation state of mTOR and its downstream substrate p70S6K in the hippocampus. Then, the animals received a bilateral infusion of rapamycin (mTOR inhibitor) in the CA1 region of the hippocampus. Their results show that rats who received the infusion 15 min before training showed impairment in long-term memory without affecting short-term memory and that these rats were capable of learning the IA task in a second training session. In contrast, rats that received the infusion immediately after training showed no effect on their long-term memory retention scores. The authors also found an increase in the activation of p70S6K 15 min after training and conclude that the mTOR-p70S6K cascade is required for protein synthesis required for memory processing [72].

### 4.4. Drug-Associated Memories

Drug-associated memories persist for a long time after abstinence and this represents a core symptom of addiction. Re-exposure to drug-associated cues reactivates drug memories and triggers neuroplastic changes that promote drug-seeking behaviors. Since memory and addiction share a common neural circuitry and molecular mechanisms, clinical and laboratory studies conclude that addiction represents the pathologic hijacking of neural processes that would typically account for reward-related learning [118,119]. With this in mind, we can say that drug addiction depends on the remodeling of synapses that shape long-term memory.

In the last few years, several research studies have been published showing the role of ANLS or lactate itself in the reconsolidation of drug memories. For instance, Zang and collaborators conducted a series of experiments to determine the role of lactate transport in the reconsolidation of drug memories. They trained a group of rats for cocaine-induced conditioned place preference (CPP) or self-administration and injected DAB into the BLA immediately after retrieval. Results show that DAB injection into the BLA prevented cocaine-induced CPP expression for up to 14 days and reduced drug-seeking behavior in rats trained to self-administer cocaine. The scientists measure the lactate concentration immediately after retrieval and found a lower concentration of lactate in the BLA. The reason to choose BLA as an injection site is because the amygdala controls emotional responses, and therefore it plays a key role in encoding conditioned drug-related information. Additionally, the authors used antisense oligonucleotides to disrupt the expression of MCTs and observed that the disruption of MCT1 and MCT2 in the BLA caused deficits in the expression of cocaine-induced CPP. While lactate co-administration can rescue the effects in MCT1 it does not occur in MCT2. Finally, it is demonstrated that glycogenolysis inhibition and its consequent reduction in lactate release decreases the gene expression of pCREB, pERK and pcoffilin, which are associated with synaptic plasticity and memory reconsolidation [120].

A similar work involving DAB administration into the BLA was reported by Boury-Jamot and co-workers. They were aiming to explore the role of the ANLS for the acquisition and maintenance of cocaine-induced memories. Their findings demonstrate that inhibition of glycogenolysis prevents the acquisition of cocaine-induced CPP in a lactate-reversible manner. This manipulation also disrupts the expression of BDNF and Zif-268, which are involved in the modulation of synaptic morphology and plasticity underlying the learning processes that strengthen conditioned responses to cocaine. Moreover, the co-administration of lactate rescues drug-associated memory through a mechanism that requires Zif-268 and the ERK signaling pathway, but not BDNF. Finally, the authors conclude that the storage and retrieval of drug-associated memories require astrocyte-derived lactate [121].

Another study that reflects the role of BLA in the reconsolidation of cocaine-associated memories was carried out by Wu et al. They aim to explore the role of glycogen synthase kinase 3β (GSK-3β) in this process. Their results show increased GSK-3β activity in the BLA in rats that acquired cocaine-induced CPP after a memory reactivation process. Some rats received a systemic injection of lithium chloride or SB216763 (GSK-3β inhibitors); this resulted in an impaired reconsolidation of cocaine cue memories and the consequent GSK-3β activity in the BLA. These findings indicate the importance of GSK-3β in the BLA in the consolidation of drug-associated memories [122].

Other molecules that play a key role in synaptic plasticity and memory consolidation are eukaryotic initiation factors. In a series of experiments, Jian and collaborators elucidated the role of eIF2a dephosphorylation in the BLA to reconsolidate drug-associated memories; this was done using the CPP task and self-administration procedures in rats. Their results display decreased levels of eIF2a phosphorylation and the activation transcription factor 4 (ATF4) in the BLA after memory retrieval procedure in a morphine- and cocaine-paired context. A group of animals received an intra-BLA infusion of Sal003 (eIF2a dephosphorylation inhibitor) immediately after retrieval; they showed a disruption in the reconsolidation of drug-induced CPP, leading to the suppression of stimulus-induced craving. This disruption in reconsolidation was blocked by the knockdown of ATF4 expression in the BLA [123].

## 5. Morphological Changes Associated with Memory Consolidation: Role of Lactate

The most common brain-imaging techniques to study brain energy metabolism in vivo are positron emission tomography (PET) and functional magnetic resonance imaging (fMRI). PET monitors changes in the blood flow, oxygen consumption and glucose utilization, whereas fMRI tracks the degree of blood oxygenation and flow. Furthermore, nuclear magnetic resonance (NMR) studies provide an insight into the relationship between glucose consumption and glutamate-glutamine cycling [3,78,124]. In the past few years, neuroscientists have been using high-resolution imaging techniques such as scanning electron microscopy (SEM) and transmission electron microscopy (TEM). These techniques can be adapted to novel set-ups that allow the acquisition of high-resolution image stacks. The segmentation of these image stacks results in accurate 3D models of brain structures which are further analyzed using 3D visualization tools or virtual reality set-ups. These approaches facilitate brain morphology analysis at the nanoscale and the study of relationships between energy consumption and glycogen storage [6,7,14,15,125,126].

To exhibit the extent of high-resolution imaging techniques and 3D morphological reconstructions, Vezzoli et al. recently published a work where they demonstrate the role of lactate to rescue memory in mice treated with DAB. Their findings show that mice injected with DAB have fewer synaptic spines per unit volume than control mice and that a co-injection can reverse the memory loss effect with lactate. They also observed that spine density increased after learning and that DAB prevented this increase. Finally, the authors concluded that a co-administration of lactate is sufficient to rescue the memory but not to increase the number of spines or post-synaptic density [8].

## 6. Concluding Remarks

This article has reviewed the interconnection between energy metabolism, synaptic plasticity and memory consolidation. The main message is that energy substrates are not only necessary to fluke the energy-consuming process associated with synaptic plasticity, but that lactate in particular has an additional function as a signaling molecule regulating the levels of expression of plasticity-associated genes and processes. These findings suggest that pharmacological interventions aimed at promoting lactate production by astrocytes may be useful in clinical conditions characterized by cognitive impairments such as Alzheimer’s disease.
